# Certification in Lunacy

**Published:** 1902-11-29

**Authors:** 


### CERTIFICATION IN LUNACY.

The very important question raised by Sir William Gowers last week, in his Address to the Medico-Psychological Association, affords only one illustration, among many others which could be adduced, of the extent to which the laws governing the insane have become the concrete expressions of popular ignorance and of legal fiction, and how utterly remote they are either from the facts of the cases with which they profess to deal, or from the present state of medical science in relation to them. As physiologists and pathologists we know something, if as yet it be only a little, of the operations of the nervous system and of the conditions by which those operations are liable to be disturbed; but as soon as we began to call ourselves "psychologists," we cease to know even that little, and lapse into the condition of those "metaphysicians" whose science was said by Dr. Armstrong to consist in the "art of talking grave nonsense upon subjects beyond the reach of the human understanding." It is intelligible that a neuron, either faulty in its structure or supplied with faulty blood, should follow the example of the Church of Ephesus, should fall from its first love and cease to do its first works; and it is intelligible that, within certain limits, its structure may admit of being repaired or its blood-supply of being amended, with consecutive restoration of its normal powers. The means by which such beneficial changes may be promoted fall wholly within the province of the physician, as whose kingdom the sick man's chamber has been accurately enough described; and any attempt at the regulation of these means by legal enactment, or at the restraint of some of them as criminal, implies an abysmal ignorance on the part of lawyers and legislators which finds its natural outlet in presumption. At present, according to Sir Richard Gowers, the practical state of the question is that there are a large number of people who are technically insane, and who could be "certified" by any medical practitioner as "proper to be detained and placed under care and treatment," but who, if so certified and detained, would be practically certain not to recover from their disease. If they are placed under care or treatment, without being "certified," they may and often do recover; but the persons in charge of them, whether medical or lay, are liable to fine or imprisonment if they receive payment; and it is only the existence of some who are willing to incur this risk that retains large classes of the partially insane within the limits of curability. The business of being examined and certified, the legal declaration of the fact of insanity, is sufficient, in many instances, to turn the scale of events against the patient, and to place an insurmountable barrier in the way of his or her improvement. Sir William pointed out that the whole of this difficulty might in great measure be overcome if it were sufficient that the state of such a patient should be "notified," and he expressed a hope that the Lord Chancellor would before long see his way to the introduction of a Bill for the accomplishment of this much-needed end.

The question of how to define insanity for a long period afforded a battleground for contests between a certain class of medical sentimentalists and the legal profession, with the result that, when questions relating to the practical treatment of the insane came before Parliament, the doctors went to the wall, and the lawyers were left masters of the situation, with power to carry into effect every absurd hypothesis which it might please them to entertain. The principal consequence of their work has been that the so-called "border cases" of insanity are left unrecognised and untreated, and that the increase of declared insanity, terrible as it is, is almost insignificant when compared with the increasing prevalence of a variety of forms of neurotic disorder, between which and insanity it would be extremely difficult to draw the line. Sir William Gowers tells us that the number of insane persons in England and Wales under the jurisdiction of the Commissioners, in 1859, was 36,700, and that it is now 110,700, a three-fold increase in about forty years. This increase, it must be remembered, excludes the "border cases," which are left, under present arrangements, to propagate their kind, to control the education of their children, and to diffuse without restraint their various crotchets, however injurious, by the agency of the press, the pulpit, or the platform. The most cursory glance at the course of events, at what is collectively called "anti-ism," at a good deal of the recent opposition to the Education Bill, or at many prevalent fads, political or ecclesiastical, is sufficient to convince any impartial observer that the unrecognised forms of insanity are increasing, and that their increase may easily become a source of danger to the public and to the Empire. The homely wisdom of America has long since appreciated the facts of the situation; and has bestowed the name of "cranks" upon such as pro-Boers, fanatics, antis, and others their congeners. It is high time that we should not only adopt the appellation, but that we should carry its application to a practical end. It is high time that doctors should be left with a free hand, to deal with the unrecognised forms of insanity in such a manner as may be most conducive to the cure of the afflicted.

